# SPP1 and MMP1 as key therapeutic targets of Jingfang Granule in idiopathic pulmonary fibrosis: integrated bioinformatics and machine learning analysis

**DOI:** 10.3389/fphar.2026.1739181

**Published:** 2026-06-10

**Authors:** Yifan Ren, Jingzhe Gao, Kunshuang Shen, Shan Jiang, Yaoyu Xie, Yuanyuan Wang, Xiaoran Sun, Yaoai Wang, Ning Zhang, Siju Lou, Jiaxin Ding, Bingxu Mu, Guangli Yan, Guimin Zhang, Xijun Wang

**Affiliations:** 1 State Key Laboratory of Integration and Innovation of Classic Formula and Modern Chinese Medicine, National Chinmedomics Research Center, National TCM Key Laboratory of Serum Pharmacochemistry, Metabolomics Laboratory, Department of Pharmaceutical Analysis, Heilongjiang University of Chinese Medicine, Harbin, China; 2 State Key Laboratory of Integration and Innovation of Classic Formula and Modern Chinese Medicine, Lunan Pharmaceutical Group Co., Ltd., Linyi, China

**Keywords:** bioinformatics, idiopathic pulmonary fibrosis, machine learning, molecular docking, molecular dynamics simulation

## Abstract

**Background:**

Idiopathic pulmonary fibrosis (IPF) is a progressive and fatal lung disease with limited therapeutic options. This study aims to identify potential efficacy biomarkers of Jingfang Granule (JFG) and investigate the therapeutic mechanism of its key active components against critical targets in IPF.

**Methods:**

IPF-related targets were identified through bioinformatics analysis of a public IPF dataset. Co-expressed gene modules were identified using Weighted Gene Co-expression Network Analysis (WGCNA). The JFG-PF interaction network was constructed employing protein-protein interaction (PPI) methods and functional enrichment analysis, which included Gene Ontology (GO), Kyoto Encyclopedia of Genes and Genomes (KEGG), and Gene Set Variation Analysis (GSVA). Additionally, five types of machine learning techniques were utilized to obtain diagnostic markers, which were further analyzed in immune infiltration assessments to evaluate their associations with immune cells and potential therapeutic effects. Molecular docking and molecular dynamics simulations were conducted to validate these analytical results. Finally, immunohistochemistry and immunofluorescence were used for verification.

**Results:**

A total of 3, 360 upregulated and 240 downregulated differentially expressed genes (DEGs) were identified in IPF sample. Integration of WGCNA findings with 1, 077 targets of JFG pinpointed 57 key genes lingking with IPF and JFG. Machine learning algorithms further refined this list, identifying four diagnostic markers:SPP1, MMP1, AKR1B10, and HTR2A. Immune infiltration analysis revealed that these biomarkers are significantly correlated with alterations in multiple immune cell populations within the IPF microenvironment. Molecular docking experiments strong binding affinities between the active compounds of JFG and these protein biomarkers. Subsequent *in vitro* validation results demonstrated that JFG significantly regulates the expression of SPP1 and MMP1 in lung fibroblasts, thereby attenuating fibrotic responses through the suppression of pro-fibrotic pathways.

**Conclusion:**

The study identified four key genes as potential diagnostic markers for IPF and therapeutic targets for JFG. The finding preliminarily elucidate ther mechanisms of JFG in mitigating pulmonary fibrosis vis regulation of fibrotic pathways and the immune microenvironment, thereby providing an integrativeevidence chain for the development of novel-fibrotic therapeutic.

## 1 Introduction

Idiopathic Pulmonary Fibrosis (IPF) is a progressive and fatal interstitial lung disease characterized by irreversible pulmonary fibrosis, with a median survival of only 2–5 years post-diagnosis (Honda et al., 2023; Fujimoto et al., 2015). With the intensification of population aging, the global burden of this disease continues to increase. Its core pathogenesis is attributed to abnormal repair responses triggered by repeated micro-injuries to the alveolar epithelium in genetically susceptible individuals (Jiang et al., 2025; Selman et al., 2019; MacNee et al., 2014). In this process, the dysregulation of repair mechanisms leads to the persistent activation of fibroblasts and myofibroblasts (Jiang et al., 2025). Resulting in excessive deposition of extracellular matrix and the formation of fibrous scars, ultimately leading to progressive destruction of lung tissue structure (Pan et al., 2025; Fijardo et al., 2024). Recent studies have revealed that immune dysregulation plays a crucial role in the pathogenesis of IPF, with aberrant activation of both the innate and adaptive immune systems involved in the entire process of epithelial injury, fibroblast activation, and ECM remodeling. The complex interactions between immune cells and structural cells create a self-perpetuating fibrotic microenvironment. Current treatment strategies for IPF include anti-fibrotic drugs such as nintedanib and pirfenidone, which can partially slow the decline in lung function (Gao et al., 2024). However, these treatments still face challenges, including (Glass et al., 2022; Chianese et al., 2024).

Currently, Traditional Chinese Medicine (TCM) is widely accepted in clinical practice due to its characteristics of multiple components, multiple targets, and minimal side effects, particularly demonstraes unique advantages in regulating the immune system and treating chronic inflammatory diseases (Abebaw et al., 2025; Reddy and Fan, 2022). Jingfang Granule (JFG) is a modern preparation derived from Jingfang Baidu San, as recorded in the Ming Dynasty herbal formulary “Shesheng Zhongmiao Fang”. This medicine has been included in the Class B of the National Reimbursement Drug List (2021 Edition). It is consists of eleven herbs:Jingjie (Schizonepeta tenuifolia (Benth.)Briq), Fangfeng (Saposhnikovia divaricate (Turcz.)Schischk), Qianghuo (Notopterygium incisum), Chaihu (Bupleurum chinense DC), Qianhu (Peucedanum praeruptorum Dunn), Chuanxiong (Ligusticum striatum DC), Zhiqiao (Aurantii Fructus L), Fuling (Poria cocos (Schw.)Wolf), Jiegeng (Platycodon grandiflorus (Jacq.)A. DC) and Gancao (Glycyrrhiza uralensis Fisch. Ex DC), JFG is utilized in the onset of new pneumonia and is clinically applied in the treatment of various epidemic and infectious diseases, including acute viral upper respiratory infections, dengue fever, influenza A (H1N1), chickenpox, and mumps (Zhu et al., 2022; Li et al., 2023). Previous studies have demonstrated that JFG exhibits significant therapeutic effects on CCl4-induced liver fibrosis, potentially related to the reduction of pro-inflammatory factor expression, enhanced antioxidant activity, and modulation of the TGF-β/Smad4 signaling pathway (Yue et al., 2025). Additionally, JFG can alleviate bleomycin-induced acute lung injury by inhibiting the NF-κB signaling pathway and promoting the generation of regulatory T cells (Tregs). Preliminary animal experiments suggest that the polysaccharide components in JFG may mitigate fibrosis by suppressing the TGF-β1/Smad3 pathway and reducing collagen deposition (Jiao et al., 2025; Yao et al., 2019; Chen et al., 2024). Furthermore, volatile oil components in JFG, such as perilla oil, may regulate the immune microenvironment and protect alveolar epithelial cells (Igarashi and Miyazaki, 2013; Yi et al., 2025). These findings indicate that JFG has potential therapeutic effects on IPF. However, the specific efficacy of JFG in treating pulmonary fibrosis, as well as its active components, molecular targets, pathway mechanisms, and clinical evidence in patients with IPF, necessitate further in-depth research.

To address the aforemidentionfied gap, this study aims to systematically identify the key therapeutic targets of JFG in anti-fibrosis and elucidate its molecular mechanisms of action. We employed integrated bioinformatics analysis of IPF gene expression profiles to screen for differentially expressed genes and co-expression modules. This analysis was combined with JFG component target identification to uncover shared co-expression gene modules between the drug and the disease. Subsequently, we utilized five machine learning algorithms to screen core diagnostic biomarkers from the intersecting targets. Finally, we conducted further validation through immune infiltration, molecular docking, and molecular dynamics simulations. The findings reveal the key targets and pathways through which JFG alleviates pulmonary fibrosis from multiple dimensions, providing a mechanistic basis for its clinical application.

## 2 Materials and methods

### 2.1 Data collection

The IPF dataset utilized in this study was acquired from the GEO database (https://www.ncbi.nlm.nih.gov/geo/) using the search keyword’pulmonary fibrosis’. The resulting dataset, GSE10667, comprises lung tissue samples from 23 stable IPF patients, 8 patients with acute exacerbation of IPF, and 15 normal controls from disease-free tissues (Rosas et al., 2008). The second dataset, GSE53845, includes lung tissue samples from 40 IPF patients and 8 healthy controls {DePianto, 2015 #3563}. These two datasets constitute the basis for the subsequent differential expression analysis and bioinformatics mining in this study.

### 2.2 Identification of differentially expressed genes

**FIGURE 1:**
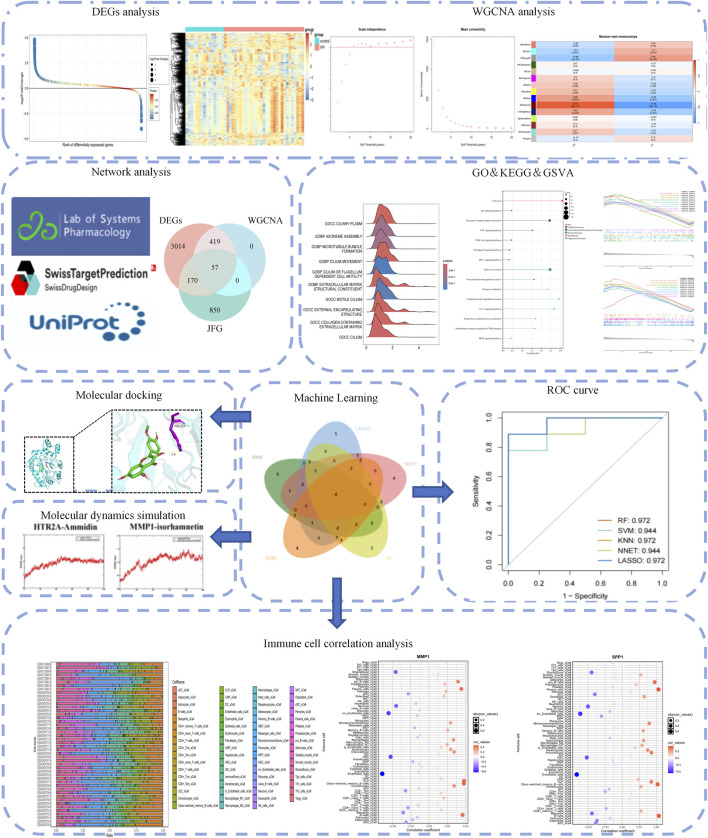
The complete research workflow.

The raw microarray data were normalized and subjected to quality control using the limma package (version 3.4.6) in R (version 4.4.3). Differential expression analysis was conducted based on the criteria of adjusted p < 0.05 and|log2 fold change (FC)| ≥1.2 to identify differentially expressed genes (DEGs) (Love et al., 2014). Significantly differentially expressed genes were visualized through volcano plots and hierarchical clustering heatmaps. Functional enrichment analysis was performed on DEGs using the clusterProfiler package (version 3.14.3) along with the org. Hs. e.g., db annotation (version 3.1.0) to identify significantly enriched Gene Ontology (GO) terms and KEGG pathways (p < 0.05) (Li and Maierheba, 2024). A brief workflow of this study is illustrated in Figure 1.

### 2.3 Weighted gene co-expression network analysis

To investigate the association between gene expression levels and diseases, we constructed a co-expression network using the “WGCNA” package (version 1.71) (Love et al., 2014). Following hierarchical clustering to eliminate outlier samples, genes exhibiting a median absolute deviation of expression variability ranking in the lowest 50% were excluded from the analysis. Based on the threshold established by the pickSoftThreshold function, the minimum power that achieved a scale-free topology fit index (R^2^) greater than 0.85 was identified, leading to the construction of an unsigned scale-free network. The adjacency matrix was then converted into a Topological Overlap Matrix (TOM), and gene modules were delineated through dynamic pruning (with a minimum module size of 30). Modules exhibiting eigengene dissimilarity of less than 0.25 (correlation coefficient >0.75) were merged. The relationship between modules and phenotypes was quantified by calculating Gene Significance (GS) and Module Membership (MM). The grey module (comprising unclassified genes) was excluded from all subsequent analyses.

### 2.4 Acquisition of Jingfang Granule targets

Eleven components of JFG, namely, Schizonepeta, Saposhnikovia, Bupleurum, Peucedanum, and other Chinese medicinal herbs, were retrieved from TCMSP (version 2.3; https://www.tcmsp-e.com/) (Ru et al., 2014). Based on the pharmacokinetic parameters provided by TCMSP, filtering criteria of OB ≥30% and DL ≥0.18 were applied to identify the qualified active ingredients from these herbal components (Xu et al., 2012). Searched for the target proteins corresponding to each active ingredient. To identify eligible active ingredients not included in TCMSP, we also consulted the literature and obtained standard SMILES structures and bioassay-confirmed targets from the PubChem database (https://pubchem.ncbi.nlm.nih.gov) (Maphetu et al., 2022). These SMILES numbers were submitted to the SwissTargetPrediction database (http://www.swisstargetprediction.ch) to predict potential target proteins of bioactive components (p > 0.4). Finally, utilize the ID mapping function of the UniProt database to standardize and deduplicate the target genes, integrate target information from all sources, and ultimately obtain the complete drug target set of JFG.

### 2.5 Construction of protein-protein interaction (PPI) network and drug–disease networks

The previously described DEGs, IPF-related targets from WGCNA, and predicted drug targets of JFG were intersected using the Venny 2.1 tool (https://bioinfogp.cnb.csic.es/tools/venny/) to identify the core therapeutic targets of JFG for IPF treatment, which were reserved for subsequent functional validation.

The resulting cross-targets were then used to construct a PPI network within the context of JFG-IPF interactions via the STRING database (https://string-db.org/) (Szklarczyk et al., 2019) with the organism limited to *Homo sapiens* and a combined score threshold of ≥0.7. The entire network, consisting of all interacting proteins that met the confidence threshold, was retained. Finally, the PPI graph was visualized using the network analyzer plugin in Cytoscape 3.9.0, thus constructing the JFG-IPF network map (Shannon et al., 2003; Assenov et al., 2008).

### 2.6 Functional enrichment analysis

To explore the potential biological functions and major signaling pathways of JFG in treating IPF, we conducted GO (Wu et al., 2021a) functional and KEGG pathway enrichment analyses on the intersection targets using the clusterProfiler package in R software (version 4.4.3) (Dennis et al., 2003; Chang et al., 2025). Both GO and KEGG analyses utilized significance thresholds of p-value<0.05. This analysis framework offers critical insights into the functional roles and molecular pathways of IPF.

### 2.7 Machine learning to identify target hub genes

To identify hub genes among the intersecting targets, five machine learning algorithms—Least Absolute Shrinkage and Selection Operator (LASSO), Random Forest (RF) (Hu and Szymczak, 2023), Support Vector Machine-Recursive Feature Elimination (SVM-RFE), K-Nearest Neighbors (KNN) (Sanz et al., 2018), and NeuroEvolution of Augmenting Topologies (NEAT)-were employed. Overlapping genes identified by all five algorithms were subsequently defined as core therapeutic targets of JFG for treating pulmonary fibrosis. Further gene correlation analysis and differential expression validation were conducted to elucidate their potential functions and clinical significance.

### 2.8 External dataset validation

To achieve cross-species analysis and further validate the research findings, this study employed the “homologene” package in R software to perform homologous conversion of hub genes into their corresponding mouse genes. Following the conversion, the expression levels of these hub genes were validated using external datasets. This validation process is crucial for confirming the relevance and importance of the identified hub genes across different biological contexts, thereby enhancing the credibility and robustness of the study results.

### 2.9 Immune infiltration analysis

To uncover the potential connections between diagnostic markers and immune cells, the R package ‘GSVA’ (version 1.44.0) was employed to estimate immune cell infiltration using the gene signature-based method xCell, This approach explored the relationship between varying levels of immune cell infiltration and the diagnostic markers for IPF. Furthermore, the correlations between hub genes and immune cells were assessed through Spearman correlation.

### 2.10 Analysis of gene set enrichment analysis for single genes

The present study employed single-gene gene set enrichment analysis (GSEA) to elucidate the functional roles of the screened biomarkers in the IPF conditions and to identify their putative downstream targets. Samples were stratified into high-and low-expression groups based on the median expression levels of the two genes of interest. The “c2.cp.kegg_legacy.v2025.1. Hs. symbols. gmt” gene set database was used as the reference standard.

### 2.11 Molecular docking

The structural information of the target hub protein was obtained from the PDB database (https://www.rcsb.org/), while the structure of the active ingredient was sourced from the PubChem database (Kim et al., 2021). These structural files underwent preprocessing utilizing AutoDock software (version 4.2.6), which included the removal of water molecules and the addition of hydrogen atoms, followed by conversion into the PDBQT format (Burley, 2025). Subsequently, we analyzed the binding sites of the target hub protein to identify the corresponding docking active pockets. The preprocessed protein and ligand files were imported into AutoDock Vina (version 1.1.2) for docking validation. The output results were then visualized in a heatmap to illustrate the potential binding affinities of these key bioactive compounds with the target hub protein. Finally, several representative docking diagrams were rendered using PyMOL (version 1.7.0; https://pymol.org/).

### 2.12 Molecular dynamics simulation

Molecular dynamics (MD) simulations were conducted using GROMACS version 2023.4. The CHARMM36 all-atom force field was employed for modeling the protein receptor, while the TIP3P model was utilized for the explicit representation of water molecules (Loubet et al., 2014). The ligand topology and parameters were generated using the CHARMM Generalized Force Field (CGenFF) through the CGenFF online server (https://cgenff.umaryland.edu/). The systems were solvated in a cubic water box, ensuring a minimum distance of 1.2 nm between the solute and the box edge. Counterions (Na+/Cl−) were introduced to neutralize the system’s charge and to establish a physiological salt concentration of 0.15 M (Milla, 2022). The system’s energy was initially minimized using the steepest descent algorithm until the maximum force dropped below 1,000 kJ/mol/nm. Following this, a two-step equilibration process was implemented:first, a 100 ps NVT equilibration at 300 K utilizing the V-rescale thermostat, and second, a 100 ps NPT equilibration at 1 bar employing the Parrinello-Rahman barostat. During both equilibration phases, position restraints were applied to the protein’s heavy atoms (da Fonseca et al., 2024). Finally, the binding free energy of the protein-ligand complex was calculated using the MM/PBSA method to evaluate its binding affinity.

### 2.13 Animal experiment

The animal protocol used in this study was approved by the Ethics Committee of the Laboratory Animal Center at Heilongjiang University of Traditional Chinese Medicine (Resolution No. 2025050935). Male C57BL/6 mice (8 weeks old, 20–25 g) were obtained from Beijing Viton–Lever Laboratory Animal Technology Co., Ltd. (Beijing, China) (SCXK–(Beijing) 2021–0006) and housed under controlled conditions (20 ± 2 °C, 40–70% humidity, 12–hour light/dark cycle) with free access to food and water. After a one-week acclimatization period, 60 mice were randomly divided into six groups (10 mice per group): control group, BLM group, BLM+JFG (low, medium, and high dose) groups, and BLM+PFD group. For intratracheal instillation, mice were anesthetized via intraperitoneal injection of sodium pentobarbital (90 mg/kg). The depth of anesthesia was confirmed by the absence of a pedal withdrawal reflex. The control group received a single intratracheal instillation of 0.9% saline, while all other groups received an intratracheal injection of bleomycin (BLM, 5 mg/kg) to induce pulmonary fibrosis (Gul et al., 2023). Starting on the day of BLM/saline administration, mice were administered JFG (1.01, 2.01, and 3.03 g/kg/day) or pirfenidone (PFD, 300 mg/kg/day) orally twice daily for 21 consecutive days.

All mice were humanely euthanized within 24 hours of the final dose. Euthanasia was performed via intraperitoneal injection of a lethal dose of sodium pentobarbital (150 mg/kg). Death was confirmed by cardiac arrest and loss of corneal reflex, followed by cardiac perfusion with pre–chilled PBS to clear residual blood from the lungs. The lungs were then excised; the left lung was used for histopathological examination, and the right lung was rapidly frozen at –80 °C for subsequent analysis. All anesthesia and euthanasia procedures were performed in accordance with the AVMA Guidelines for the Euthanasia of Animals (2020 edition).

### 2.14 Morphological analysis

The isolated lung tissues of mice were fixed in 4% polyformaldehyde for 24 h. After fixation, the tissues were embedded in paraffin and sectioned into 5 μm thick slices. The sections were stained with hematoxylin-eosin (HE) staining kit, and histopathological changes were observed under optical microscopy, with particular attention to inflammatory infiltration, structural alterations, and collagen deposition.

### 2.15 ELISA measurement of hydroxyproline (HYP)

The collagen content in mouse lung tissues was assessed using a Hydroxyproline Assay Kit (Nanjing Jiancheng Bioengineering Institute, China). Approximately 30 mg of lung tissue was accurately weighed and hydrolyzed according to the manufacturer’s protocol. The hydrolyzed samples were neutralized by adjusting the pH to a range of 6–8 to ensure optimal detection conditions. After centrifugation, the supernatant was collected, and the absorbance was measured at a wavelength of 550 nm using a spectrophotometer. The hydroxyproline content was quantified based on a standard curve to evaluate the degree of collagen deposition in the lung tissues, reflecting the extent of pulmonary fibrosis.

### 2.16 ELISA measurement of inflammatory factors

The levels of inflammatory factors in mouse lung tissues were assessed using detection kits for IL-6, IL-1β, and IL-10 (Nanjing Jiancheng Bioengineering Institute, China). Approximately 30 mg of lung tissue was accurately weighed. Tissue homogenates were prepared by homogenization and lysis according to the manufacturer’s instructions. The pH of the samples was adjusted to the range of 6.0–8.0 to meet the subsequent detection conditions. After centrifugation, the supernatant was collected, and its absorbance was measured at a wavelength of 550 nm using a spectrophotometer. The concentrations of IL-6, IL-1β, and IL-10 in the samples were calculated using standard curves to evaluate pulmonary inflammation levels, thereby reflecting the progression of pulmonary fibrosis.

### 2.17 Immunohistochemistry (IHC) and immunofluorescence (IF)

For both IHC and IF analyses, serial 4 μm paraffin-embedded tissue sections were subjected to a standardized procedure. Briefly, sections were baked at 70 °C for 2 h, deparaffinized in xylene, and rehydrated through a graded alcohol series. Antigen retrieval was performed using citrate buffer (pH 6.0). For IHC staining specifically, endogenous peroxidase activity was blocked with 0.3% hydrogen peroxide. All sections were then blocked with 2.5% sheep serum to minimize non-specific binding and subsequently incubated with primary rabbit antibodies against Collagen I (67288-1-Ig, Proteintech, Wuhan, China, 1:5,000), α-SMA (14395-1-AP, Proteintech, Wuhan, China, 1:2000), SPP1 (HA723601, HUABIO, China) and MMP1 (ER31211, HUABIO, China) at 30 °C for 80 min. For IHC detection, the sections were incubated with an HRP-conjugated secondary antibody, visualized with DAB chromogen, counterstained with hematoxylin, and mounted with neutral gum. For parallel IF detection, the sections were instead incubated with an Alexa Fluor 488-conjugated goat anti-rabbit secondary antibody, with nuclei counterstained using DAPI, and finally mounted with an anti-fade mounting medium.

### 2.18 Cell culture and processing

The human fetal lung fibroblast cell line MRC-5 was obtained from Wuhan Punosai Life Sciences Co. The cells were cultured at 37 °C in a humidified incubator with 5% CO2 using MRC5 cell-specific medium. Cells were allowed to grow to 60%–70% confluence and then serumstarved in a serum-free medium for 6–8 h. Recombinant human TGF- β1 (10 ng/mL, PeproTech, USA) was added for 24 h to stimulate the cellular lung fibrosis model.

### 2.19 Cell viability

Cell proliferation was assessed using the Cell Counting Kit-8 (CCK-8, Dojindo, Kumamoto, Japan) according to the manufacturer’s instructions. Briefly, MRC-5 cells were inoculated into 96-well plates, stimulated with TGF-β1, and treated with increasing concentrations of JFG. Cells were incubated with CCK-8 dissolved in the medium for 3 h at 37 °C, and the absorbance at 450 nm was measured using an enzyme reader (Thermo MMLTISKAN GO, USA).

### 2.20 Western blot analysis

After cell lysis, total proteins were extracted using RIPA lysis buffer (C1053, Prilosec, Beijing, China), and protein concentration was determined by BCA protein assay kit (Cat. No. P1511, Prilosec, Beijing, China). According to the molecular weight differences of target proteins, SDS-PAGE electrophoresis separation was conducted using 10% polyacrylamide gel (24 μg protein per lane), followed by transfer onto PVDF membranes. Due to the wide molecular weight range of target proteins (36–139 kDa), each target protein was analyzed on independent membranes to ensure optimal separation and detection sensitivity. After blocking with rapid blocking buffer at room temperature for 20 min, the membranes were incubated overnight at 4 °C with the following primary antibodies: Collagen I (67288-1-Ig, Proteintech, Wuhan, China, 1:5,000), E-cadherin (20874-1-AP, Proteintech, Wuhan, China, 1:5,000), ACSL4 (22401-1-AP, Proteintech, Wuhan, China, 1:5,000), SPP1 (HA723601, HUABIO, China, 1:2000), Elastin (15257-1-AP, Proteintech, Wuhan, China, 1:1,000), Occludin (27260-1-AP, Proteintech, Wuhan, China, 1:1,000), MMP1 (ER31211, HUABIO, China, 1:2000), Vimentin (10366-1-AP, Proteintech, Wuhan, China, 1:5,000), α-SMA (14395-1-AP, Proteintech, Wuhan, China, 1:2,000). To ensure that each target protein had its own specific loading control from the same membrane, the antibodies on each membrane were stripped using an enhanced stripping buffer (antibody stripping solution, enhanced) after target protein detection. The same membranes were then reprobed with GAPDH antibody (60004-1-Ig, Proteintech, Wuhan, China, 1:5,000) and visualized following the same procedure. Band density was quantified using ImageLab software (Bio-Rad), with target protein expression levels normalized to the GAPDH signal obtained from the identical membrane after stripping and reprobing.ormalized to the specific internal reference GAPDH control from the same blot experiment.

### 2.21 Statistical analyses

All data processing and analysis were conducted using R software (version 4.4.3) unless otherwise stated. p < 0.05 was considered statistically significant, and the significance level is denoted as follows: *(p < 0.05), **(p < 0.01), and ***(p < 0.001) by one-way ANOVA.

## 3 Results

### 3.1 3600 shared dysregulated genes were identified in IPF

**FIGURE 2:**
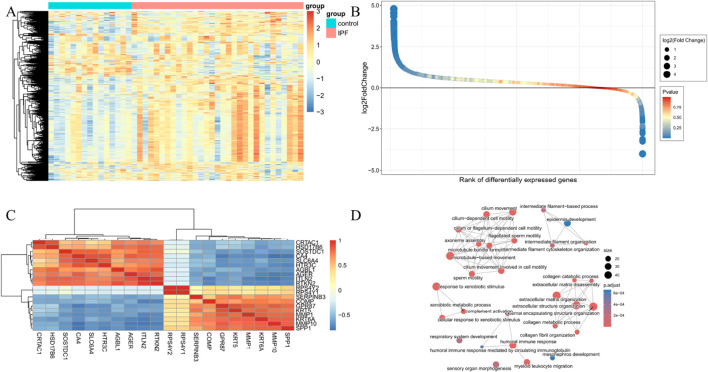
Acquisition of IPF DEGs in the GSE10667 dataset. **(A)** Heatmap of DEGs analysis results. **(B)** Volcano plot of DEGs analysis results. **(C)** Correlation among the top 20 DEGs. **(D)** Network topology of the top 20 DEGs.

Identifying 3,360 upregulated and 240 downregulated DEGs (Supplementary Table S1). The distribution of DEGs was visualized through a volcano plot (Figure 2A) and a hierarchical clustering heatmap (Figure 2B). Subsequently, a gene correlation network was constructed among the key DEGs (Figure 2C), further illustrating the distinct expression patterns of differential genes between the disease group and the control group. Network topology analysis revealed strong correlations among the pathways enriched by these differential genes (Figure 2D). All data were sourced from the publicly available GEO database, thus exempting this study from additional ethical review.

### 3.2 The darkred module, harboring 476 genes, is identified as a key co-expression unit in IPF

**FIGURE 3:**
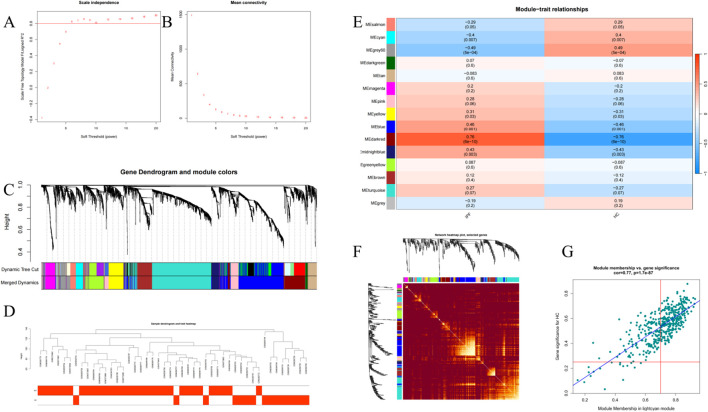
Construction of the weighted co-expression network related datasets in IPF from the GSE10667 dataset and identification of associated modules. **(A,B)** Network topology analysis for various soft thresholds. The left panel shows the scale-free fit index (scale-free, y-axis) as a function of the soft threshold power (x-axis); the right panel shows the mean connectivity (degree, y-axis) as a function of the soft threshold power (x-axis). **(C)** Gene dendrogram obtained by average linkage hierarchical clustering. The colored row below the dendrogram indicates the module assignments determined by the dynamic tree cut method. **(D)** Heatmap visualization of the clustering results of samples and phenotypic information. **(E)** Module-trait relationship analysis plot for 15 modules. Each row in the heatmap corresponds to an ME, and each column corresponds to a clinical feature. Each cell contains the corresponding correlation and p-value. **(F)** Network heatmap of correlations among module genes. **(G)** Scatter plot of IPF GS and MM in the dark red module.

Based on scale-free topology and mean connectivity analysis, the optimal soft threshold was determined to be 6 (Figures 3A,B). At this threshold, the network exhibited significant scale-free topology characteristics:the connectivity distribution showed right-skewedness (Supplementary Figure S1), and the double logarithmic coordinate plot showed good fitting (R^2^ = 0.84, k = −1.2) (Supplementary Figure S2). After merging similar modules, a dendrogram containing 15 distinct gene modules was ultimately obtained (Figure 3C). The gene co-expression modules showed significant correlations with clinical traits (Figure 3D). Furthermore, based on the gene co-expression network, a network heatmap illustrating gene connectivity within each module was generated (Figure 3E). The darkred module (i.e., MEdarkred) showed the most significant correlation with idiopathic pulmonary fibrosis/non-idiopathic pulmonary fibrosis control phenotypes (Figure 3F; Supplementary Table S2). Further analysis revealed a significant positive correlation between gene significance (GS) and module membership (MM) in the darkred module (Figure 3G). Based on these findings, the 476 genes in the darkred module were considered potential IPF-related targets for subsequent analysis.

### 3.3 Identification of 57 core therapeutic targets for JFG against IPF

**FIGURE 4:**
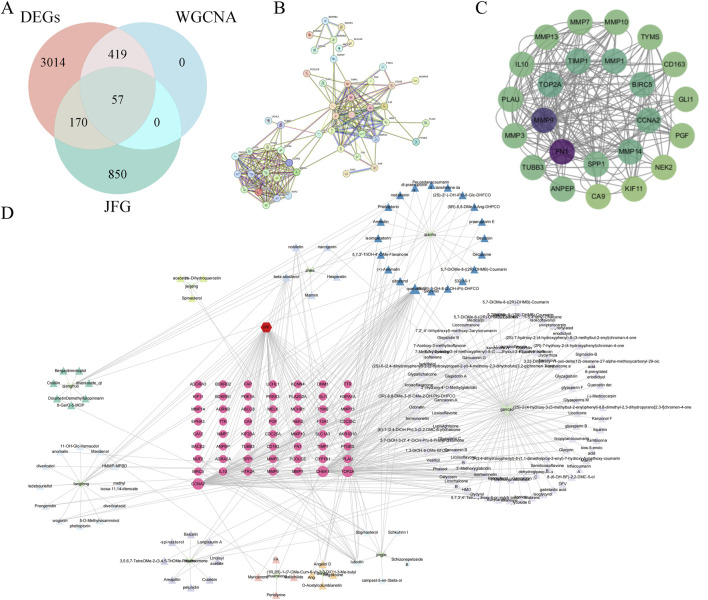
Prediction of potential targets of JFG in IPF treatment and construction of PPI and drug-disease network. **(A)** Venn diagram of potential target prediction. **(B)** Original PPI network of intersecting targets. **(C)** Visualized PPI network of intersecting targets. **(D)** JFG-IPF network diagram. The red polygon represents IPF. Triangles represent the active components of JFG. Pink circles represent the intersecting targets. Connecting lines indicate the associations between nodes.

Based on the TCMSP and SwissTargetPrediction databases, 1,077 predicted targets of JFG active ingredients were identified. Intersection with IPF DEGs and WGCNA key module genes yielded 57 overlapping genes (Figure 4A; Supplementary Table S3). Protein–protein interaction (PPI) network analysis revealed targets with high degree values as potentially critical nodes (Figure 4B; Tables 1, 2). The study constructed a component-target-disease network, elucidating the multi-target regulatory potential of JFG against IPF (Figures 4C,D). Ultimately, these 57 core targets were screened for subsequent functional validation.

**TABLE 1:** Information on the top 20 targets sorted by degree value.

Full target name	Target acronym	Degree	Betweenness	Closeness
Fibronectin 1	FN1	42	650.5849	0.55813956
Matrix metallopeptidase 9	MMP9	38	616.08093	0.54545456
DNA topoisomerase II alpha	TOP2A	30	245.22382	0.46601942
TIMP metallopeptidase inhibitor 1	TIMP1	30	107.54682	0.46153846
Matrix metallopeptidase 1	MMP1	24	80.096825	0.46153846
Baculoviral IAP repeat containing 5	BIRC5	30	228.92857	0.45714286
Cyclin A2	CCNA2	28	166.88095	0.4528302
Matrix metallopeptidase 14	MMP14	20	58.480953	0.4528302
Secreted phosphoprotein 1	SPP1	32	78.153175	0.44036698
Tubulin beta 3 class III	TUBB3	14	144.68571	0.42857143
Alanyl aminopeptidase	ANPEP	8	574	0.42857143
Plasminogen activator, urokinase	PLAU	26	13.93254	0.42105263
Interleukin 10	IL10	24	23.9	0.42105263
Matrix metallopeptidase 3	MMP3	24	6.811111	0.42105263
Matrix metallopeptidase 7	MMP7	22	74.10714	0.41379312
Matrix metallopeptidase 13	MMP13	22	1.4444444	0.41379312
Matrix metallopeptidase 10	MMP10	18	12.476191	0.41025642
Thymidylate synthetase	TYMS	30	80.94762	0.40677965
CD163 molecule	CD163	16	102.5	0.4
GLI family zinc finger 1	GLI1	8	8.571428	0.3966942

**TABLE 2:** Information on the top 20 active ingredients sorted by the degree value.

Ingredient code	Ingredient name	Degree	Betweenness centrality	Closeness centrality
Qianhu	Quercetin	44	0.098517338	0.417142857
Jingjie	Luteolin	16	0.030581859	0.323485968
Zhike	Beta-sitosterol	12	0.039890223	0.317851959
Jingjie	Stigmasterol	8	0.007539193	0.289299868
Gancao	Kaempferol	8	0.01590294	0.380869565
Zhike	Nobiletin	6	0.018997102	0.345971564
Qianhu	Sitosterol	6	0.090189889	0.358428805
Gancao	Isorhamnetin	6	0.015982971	0.364392679
Qianhu	(+)-Anomalin	4	0.008665832	0.326378539
Qianhu	Ammidin	4	0.005454494	0.271375465
Qianhu	Isoimperatorin	4	0.005454494	0.271375465
Gancao	Lupiwighteone	4	0.003521152	0.375643225
Gancao	Glyasperin C	4	0.003521152	0.375643225
Gancao	Kanzonols w	4	0.003521152	0.375643225
Gancao	Glepidotin A	4	0.003521152	0.375643225
Gancao	Gancaonin A	4	0.003521152	0.375643225
Fangfeng	Wogonin	4	0.011070485	0.330316742
Fangfeng	Prangenidin	3	0.010132485	0.341653666
Chuanxiong	Myricanone	3	0.016696489	0.328335832
Qianhu	Decursin	2	9.07E-04	0.262905162

### 3.4 Functional enrichment analysis reveals potential biological processes and pathways associated with the candidate targets

**FIGURE 5:**
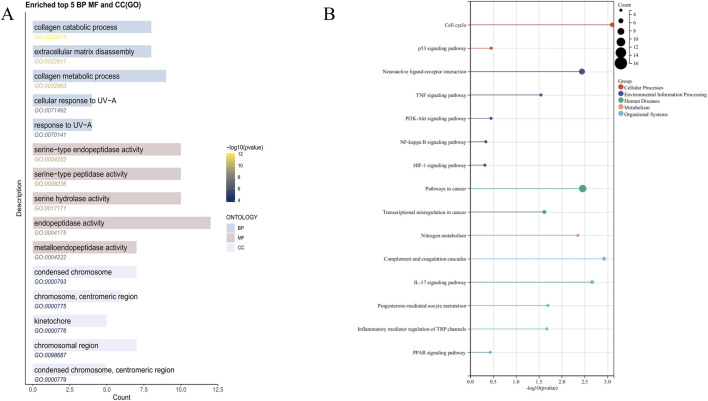
Functional enrichment analysis of intersection genes in IPF. **(A)** Bar plot of GO analysis. **(B)** Lollipop plot of KEGG analysis.

GO and KEGG pathway enrichment analyses revealed that 57 cross-target genes were significantly enriched in cell cycle regulation and chromosome dynamics. Key Biological Processes (BP) included mitotic nuclear division, G2/M transition, spindle organization, sister chromatid separation and chromosome segregation, as well as collagen catabolic processes, UV response, and wound healing. Cellular Components (CC) emphasized the localization of chromosome kinetochore structures and spindle microtubules. Molecular function (MF) indicated associations with serine-type endopeptidase activity and collagen/microtubule binding capacity (Figure 5A). KEGG analysis further revealed that the differentially expressed genes were primarily involved in inflammation and immune response pathways (including the TNF signaling pathway, NF-kappa B signaling pathway, IL-17 signaling pathway, and complement and coagulation cascades), core pathways of tissue repair and fibrosis (such as the PI3K-Akt signaling pathway and HIF-1 signaling pathway), as well as cell cycle and apoptosis regulation (p53 signaling pathway). These findings suggest that the pathological progression of pulmonary fibrosis may be closely associated with recurrent alveolar epithelial injury, dysregulated inflammatory immune responses, and abnormal tissue repair mechanisms (Figure 5B).

### 3.5 SPP1, MMP1, HTR2A, and AKRB10 were identified as core biomarkers for IPF

**FIGURE 6:**
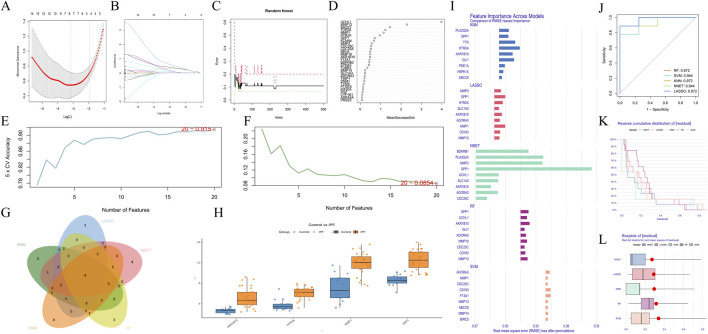
Identification of target hub genes through machine learning methods. **(A)** Cross-validation curve of LASSO analysis. **(B)** Coefficient path diagram of LASSO analysis. **(C)** Error rate curve of the RF algorithm. **(D)** Ranking of important genes predicted by the Random Forest algorithm. **(E)** Accuracy rate of the Support Vector Machine algorithm. **(F)** Error rate of the Support Vector Machine algorithm. **(G)** Venn diagram of hub genes from five machine learning methods. **(H)** Box plot of hub target gene expression analysis based on the GSE10667 dataset. **(I)** Evaluation of gene importance in each machine learning algorithm. **(J)** Box plot of residuals from five machine learning methods, with the root mean square error (RMSE) of residuals represented by red dots. **(K)** Cumulative residual distribution of five learning models. **(L)** ROC curves of four machine learning models.

This study employed five machine learning techniques to precisely identify key genes in the IPF dataset. The ability to distinguish IPF from non-IPF samples served as the evaluation criterion. Specifically, the LASSO algorithm identified 20 core genes, including BIRC5, PGF, and TYMS (Figures 6A,B); the RF method selected 20 genes such as TIMP1, TUBB3, and MMP7 (Figures 6C,D); the SVM-RFE approach identified 20 core genes, including AKR1B10, SPP1, and HSPA1A (Figures 6E,F); the KNN method screened 20 genes, including MMP9 and IL10; and the NNET method selected 20 genes, such as CDC25C, NUF2, and PGF. By intersecting the genes identified by all five methods, four overlapping genes were obtained: AKR1B10, SPP1, MMP1, and HTR2A (Figure 6G). Additionally, the box plot illustrating the intersection genes in relation to clinical information demonstrated significant differences between patients with pulmonary fibrosis and healthy individuals (Figure 6H). To evaluate the contribution of each feature gene in the model, we calculated the increase in RMSE loss for each gene across the algorithm based on the permutation test method and generated a feature importance ranking plot accordingly (Figure 6I). These findings suggest that these genes may serve as potential biomarkers associated with the pathogenesis of IPF.

The validation results of the five models revealed that all achieved AUC values above 0.9, indicating good classification performance (Figure 6J). Residual distribution analysis showed low RMSE values, suggesting minimal prediction errors (Figure 6K), and the predicted values were highly consistent with actual valuess (Figure 6L). These results suggest that the combination of these algorithms provides a robust approach for identifying IPF-associated genes.

The four gene model based on SPP1, MMP1, HTR2A, and AKR1B10 achieved an AUC of 0.988 in external validation, demonstrating excellent predictive performance.

**FIGURE 7:**
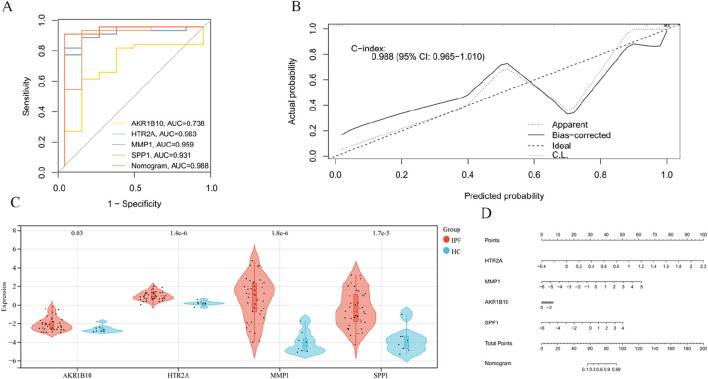
Performance Evaluation of the Prediction Model Constructed Based on SPP1, MMP1, HTR2A, and AKR1B10 in External Validation. **(A)** Evaluation of the Discriminative Ability of Prediction Models. **(B)** Model calibration curve. **(C)** Differentially expressed gene analysis. **(D)** Nomogram scoring system.

The predictive performance of the four-gene (SPP1, MMP1, HTR2A, and AKR1B10) model was evaluated using an independent external validation dataset (GSE53845). The model achieved an area under the receiver operating characteristic curve (AUC) of 0.988 (Figure 7A), with a concordance index (C-index) of 0.988 (95% CI: 0.965–1.010), indicating high predictive accuracy (Figure 7B). When analyzed individually, the AUC values were 0.963 for HTR2A, 0.959 for MMP1, 0.931 for SPP1, and 0.738 for AKR1B10 (Figure 7A). Differential expression analysis confirmed that HTR2A and MMP1 were significantly upregulated in IPF samples (median values: 0.10 and 0.20, respectively), while AKR1B10 and SPP1 were significantly downregulated (both median values: -3.5) (Figure 7C). The clinical scoring system constructed based on these genes converts the expression levels of each gene into intuitive scores: HTR2A (0–2.2 points), MMP1 (−6–5 points), AKR1B10 (0 to −2 points), SPP1 (−6–4 points), with a total score range of 0–200 points corresponding to predicted probabilities of 0.1–0.99. Calibration curve analysis showed good agreement between the model’s predicted probabilities and actual observed outcomes (Figure 7D). In summary, the four-gene predictive model demonstrated good discriminative ability and calibration in external validation, suggesting its potential utility for risk assessment in IPF.

### 3.6 Oxidative stress serves as a critical downstream mechanism through which SPP1, MMP1, HTR2A, and AKR1B1 mediate the progression of pulmonary fibrosis

**FIGURE 8:**
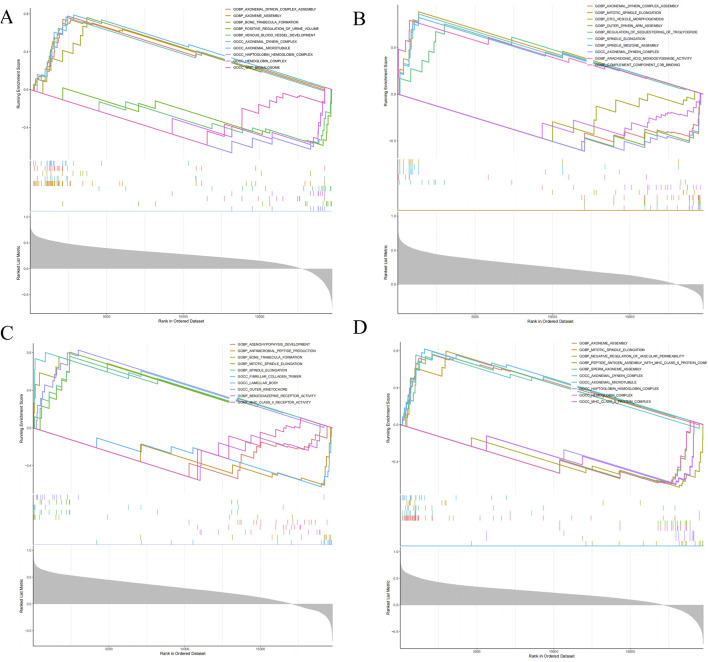
GSEA analysis of SPP1, MMP1, HTR2A, and AKRB10 in IPF. **(A)** GSEA analysis of SPP1; **(B)** GSEA analysis of MGP; **(C)** GSEA analysis of HTR2A; **(D)** GSEA analysis of AKRB10.

GSEA analysis revealed that these genes are associated with epithelial repair and communication abnormalities, involving the regulation of vascular permeability (AKR1B10), cilia assembly (SPP1, AKR1B10), and WNT signaling (SPP1) (Figures 8A,B); they are also enriched in immune-inflammatory pathways, including antimicrobial peptide production (HTR2A), MHC class II complex assembly (HTR2A, AKR1B10), and complement binding (MPP1) (Figures 8B,C). More importantly, evidence directly related to oxidative stress was identified, such as AKR1B1-mediated aldehyde detoxification (associated with AKR1B10) and hemoglobin complex formation (SPP1, AKR1B10) (Figures 8A,C). These findings suggest that oxidative stress-related pathways may represent a potential mechanism linking the expression of these genes to the progression of IPF, as further illustrated by the GSEA enrichment curves for SPP1 (Figure 8A), MPP1 (Figure 8B), HTR2A (Figure 8C), and AKR1B10 (Figure 8D).

### 3.7 Immune infiltration analysis reveals distinct immune cell composition in IPF tissues

**FIGURE 9:**
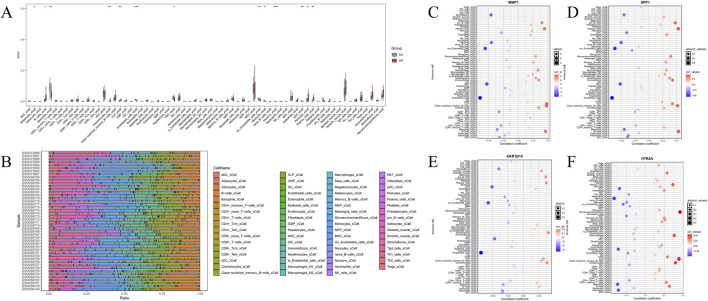
Immune infiltration analysis of hub genes. **(A)** Box plot of different immune cell infiltrations in IPF and normal samples. **(B)** Stacked bar chart of various types of immune cell infiltrations in each sample of the GSE10667 dataset. **(C–F)** Heatmap of the correlation between immune infiltration and hub gene expression.

The results revealed significant differences in the immune microenvironment between IPF tissues and the HC group (Figures 9A,B), with notably increased enrichment scores for M2 macrophages, total macrophages, and fibroblasts in IPF, and significantly decreased scores for plasma cells, CD4+naive T cells, CD8+T cells, and NK cells. Further Spearman correlation analysis indicated that endothelial cells, fibroblasts, and hematopoietic stem cells were significantly negatively correlated with the expression of four core genes, while B cells, plasma cells, and class-switched memory B cells showed significant positive correlations (Figures 9C–F).

### 3.8 Molecular docking predicts potential interactions between JFG active components and core targets

**FIGURE 10:**
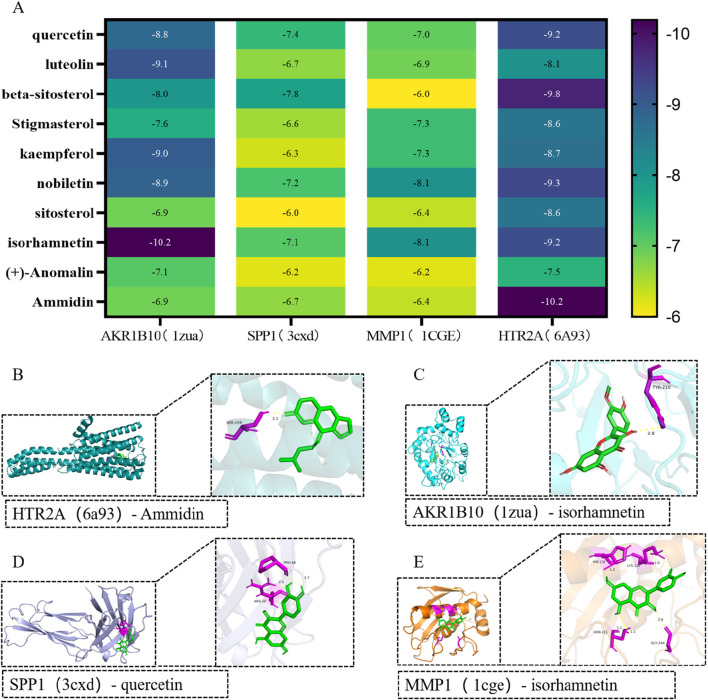
Molecular docking analysis of JFG active ingredients with target proteins. **(A)** Heatmap of binding energy (kcal·mol-1) between active ingredients and target hub proteins. **(B–E)** Docking diagrams of the highest binding energy components interacting with hub genes.

The binding potential between the active ingredients of JFG and the core target genes was assessed using molecular docking analysis. Protein structures for the core targets were obtained from the Protein Data Bank (PDB) as protein receptors, including AKR1B10 (PDB ID:1ZUA), SPP1 (PDB ID:3CXD), MMP1 (PDB ID:1CGE), and HTR2A (PDB ID:6A93). The top 10 active ingredients with the highest degree values in the network were selected as ligands: quercetin, luteolin, beta-sitosterol, stigmasterol, kaempferol, nobiletin, sitosterol, isorhamnetin, (+)-anomalin, and ammidin. A binding energy threshold of <−5 kcal/mol is generally considered to indicate potential binding affinity. The docking results showed that all selected active ingredients exhibited binding energies below this threshold with the core targets (Figure 10A), suggesting potential interactions. Representative binding modes of selected target-component pairs with strong binding affinities are illustrated in Figures 10B–E.

### 3.9 Molecular dynamics simulations predict stable interactions between JFG components and core targets

**FIGURE 11:**
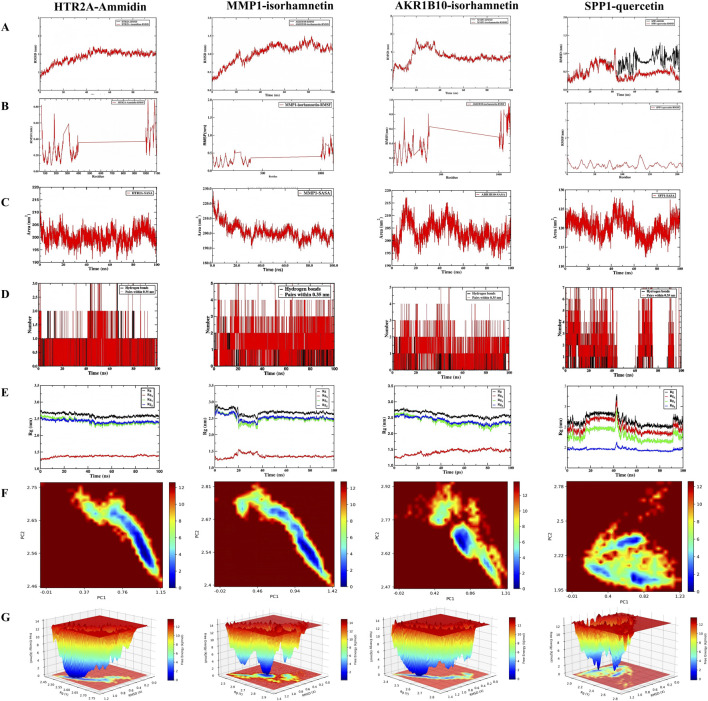
Molecular dynamics simulation of JFG active ingredients and target proteins. **(A)** Time-dependent changes in the root mean square deviation of backbone atoms between the protein and ligand. **(B)** Analysis of root mean square fluctuations for each residue of the protein and ligand. **(C)** Time-dependent changes in the total solvent accessible surface area of the protein and ligand. **(D)** Time-dependent changes in the number of hydrogen bonds between the protein and ligand. **(E)** Time-dependent changes in the radius of gyration of the protein and ligand. **(F)** 2D free energy landscape of the protein and ligand. **(G)** 3D free energy landscape of the protein and ligand.

To assess the stability of protein-ligand interactions, molecular dynamics simulations were conducted on four complexes:HTR2A-Ammidin, AKR1B10-isorhamnetin2, SPP1-quercetin, and MMP1-isorhamnetin. RMSD analysis indicated that all systems reached a stable state (<2 nm), and RMSF results showed that the conformational fluctuations of the complexes were minor, with tighter structures (Figures 11A,B). SASA decreased from 230 nm^2^ to 190 nm^2^and reached equilibrium at 60 ns, while the number and strength of hydrogen bonds remained stable throughout the process, reflecting strong binding interactions (Figures 11C,D). The radius of gyration remained stable between 2.3 and 2.5 nm, indicating good binding tightness (Figure 11E). Additionally, the 2D/3D free energy landscapes revealed the most stable conformational states of each complex system (Figures 11F,G). The MMPBSA method was employed to calculate the binding free energies of four protein-ligand complexes. The results demonstrated that the AKR1B10-isorhamnetin complex exhibited the strongest binding affinity. The AKR1B10-isorhamnetin (ΔG = −127.44 kJ/mol, Ki = 4.72 × 10^−14^ nM), MMP1-isorhamnetin (ΔG = −120.55 kJ/mol, Ki = 7.61 × 10^−13^ nM), HTR2A-Ammidin (ΔG = −117.93 kJ/mol, Ki = 2.19 × 10^−12^ nM), and SPP1-quercetin (ΔG = −73.25 kJ/mol, Ki = 1.47 × 10^−4^ nM). Among them, MMP1-isorhamnetin contributes the most to VDW (−179.01 kJ/mol). These computational predictions suggest that the JFG components may form stable complexes with the core targets, providing a theoretical basis for their potential interactions (Supplementary Figure S3).

### 3.10 JFG ameliorates pulmonary fibrosis in a bleomycin-induced mouse model

**FIGURE 12:**
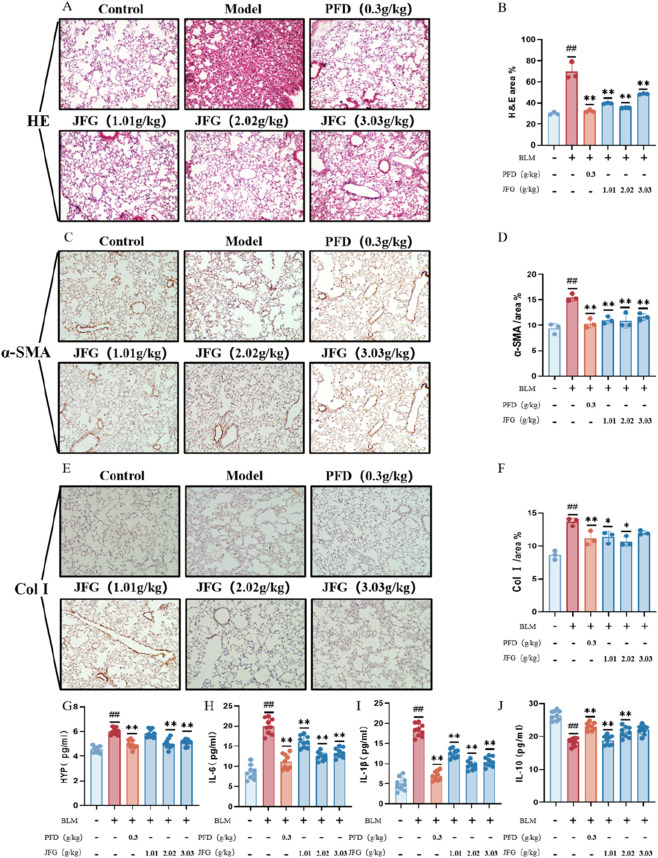
JFG can alleviate pulmonary pathology and inflammatory responses in IPF mice. **(A,B)** Haematoxylin and eosin (H&E). **(C,D)** Representative images of α-SM IHC staining in lung tissues from each group of mice. The brown-yellow signals in the cytoplasm indicate α-SMA-positive myofibroblasts. **(E,F)** Representative images of Col Ⅰ IHC staining in lung tissues from each group of mice. The brown-yellow signals in the cytoplasm indicate Col Ⅰ-positive myofibroblasts. **(G–J)** The levels of HYP and pro-inflammatory cytokines measured by ELISA (Pirfenidone (PFD), 300 mg/kg, positive control; ^#^p < 0.05, ^##^p < 0.00 vs. control group; *p < 0.05, **p < 0.01 vs. model group; n = 9); Scale bar = 50 μm.

As shown in Figures 12A,B, HE staining of lung sections revealed significant morphological damage in the MOD group, characterized by marked inflammatory cell infiltration and hemorrhage in the alveoli and airways. JFG-L, JFG-M, and JFG-H (1.01, 2.02, and 3.03 g/kg, respectively) significantly alleviated the pathological changes in lung tissue, with gradual improvement in pulmonary structure compared to the MOD group. Further immunohistochemical staining demonstrated that JFG markedly reduced collagen deposition, as evidenced by decreased positive areas of α-SMA and Collagen I in lung tissue (Figures 12C–F). The ELISA results showed that compared with the Con group, the levels of HYP, IL-6 and IL-1β in lung tissues of mice in the Mod group were significantly increased (P < 0.01), while the level of IL-10 was significantly decreased (P < 0.01). Compared with the Mod group, the levels of HYP,IL-6 and IL-1β in the Pos group, JFG-L, JFG-M, and JFG-H groups were significantly decreased (P < 0.01), and the level of IL-10 was significantly increased (P < 0.01) (Figures 12G–J). These results suggest that JFG attenuates pulmonary fibrosis in mice, potentially through modulation of inflammatory responses and reduction of collagen deposition.

### 3.11 IHC and IF analyses reveal differential expression of SPP1 and MMP1 in fibrotic lung tissues and response to JFG treatment

**FIGURE 13:**
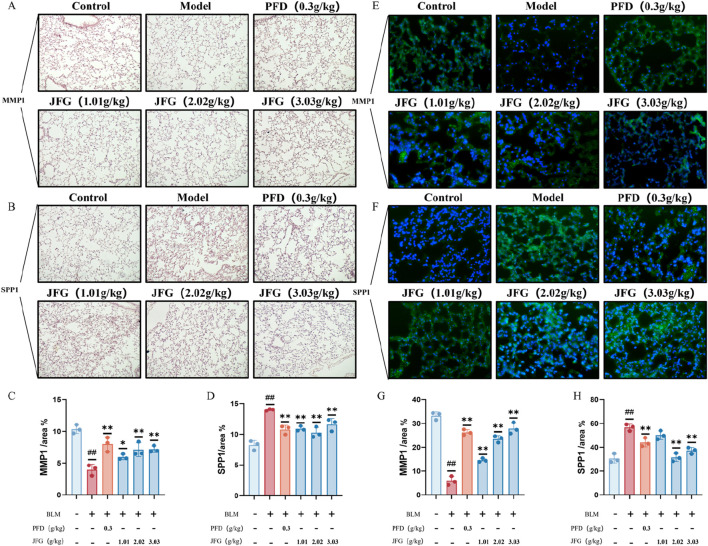
IHC and IF was employed to detect the expression levels of MMP1 and SPP1. **(A–D)** Representative images of SPP1 and MMP1 IHC staining in lung tissues from each group of mice. The brown-yellow signals in the cytoplasm indicate positive myofibroblasts. **(E–H)** Representative IF staining images of MMP1 and SPP1 in lung tissues from each group. MMP1 and SPP1 exhibited green fluorescence, localized in the cytoplasm; nuclei were counterstained with DAPI and displayed blue fluorescence. (Pirfenidone (PFD), 300 mg/kg, positive control; ^#^p < 0.05, ^##^p < 0.01 vs. control group; *p < 0.05, **p < 0.01 vs. model group; n = 3); Scale bar = 50 μm.

IHC and IF analyses yielded consistent results regarding the expression and localization of SPP1 and MMP1. Both IHC staining and IF signals demonstrated that SPP1 and MMP1 proteins were primarily localized in the cytoplasm, exhibiting brownish-yellow granular patterns and distinct fluorescent signals, respectively. As shown in Figures 13A–D, the expression profiles of SPP1 and MMP1 exhibited significant differences in fibrotic lung tissues. Consistent with its pro-fibrotic role, the model group demonstrated markedly elevated SPP1 expression levels, characterized by significantly enhanced staining intensity, bright fluorescent signals, and a substantial increase in positive cell ratio, which stood in sharp contrast to the weak expression observed in the control group. In alignment with the pathological mechanism of impaired collagen degradation in fibrosis, MMP1 expression levels were significantly reduced in the model group. Following drug intervention, these trends were reversed: both the positive expression area and intensity of SPP1 showed obvious reduction, while MMP1 expression demonstrated a significant increase (Figures 13E–H). These results indicate that SPP1 is upregulated and MMP1 is downregulated in pulmonary fibrosis, and that JFG treatment can modulate the expression of both proteins, consistent with its observed anti-fibrotic effects.

### 3.12 JFG inhibits MRC-5 cell proliferation in a concentration-dependent manner

**FIGURE 14:**
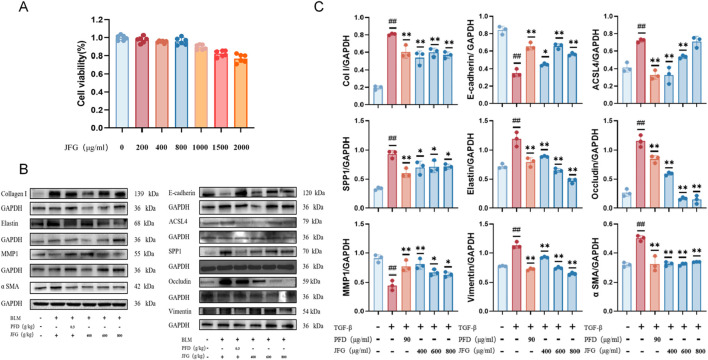
JFG inhibits MRC-5 cell proliferation and ameliorates BLM-induced fibrotic phenotypes *in vitro*. **(A)** Effects of different concentrations of JFG on the viability of MRC-5 cells. **(B,C)** Effects of JFG on the expression of fibrosis, EMT, and oxidative stress-related proteins in BLM-induced MRC-5 cells (Pirfenidone (PFD), 90 μ/mL, positive control; ^#^p < 0.05, ^##^p < 0.00 vs. control group; *p < 0.05, **p < 0.01 vs. model group; n = 3).

The CCK-8 cell proliferation assay can rapidly determine the toxicity of drugs on selected cells, facilitating the screening of appropriate dosing concentrations to better serve downstream experiments. Different concentrations of JFG were added to the human fetal lung fibroblast cell line MRC-5, and the inhibitory effect of JFG on MRC-5 proliferation was evaluated using the CCK-8 assay. The results (Figure 14A) demonstrated that JFG lyophilized powder at concentrations ranging from 400 μg/mL to 2 mg/mL effectively inhibited cell proliferation after 24 h of intervention, with a concentration-dependent effect. However, when the JFG concentration exceeded 1 mg/mL, the inhibitory effect on MRC-5 was too strong, resulting in cell viability below 80%. Based on the CCK-8 experimental results, we selected three concentrations (low, medium, and high) of JFG lyophilized powder within the range of 400 ug/mL to 800 ug/mL for subsequent cell experiments.

### 3.13 JFG modulates expression of fibrosis-, EMT-, and oxidative stress-related proteins in MRC-5 cells

To evaluate the effects of JFG on extracellular matrix deposition, epithelial-mesenchymal transition, and the expression of oxidative stress-related proteins in MRC5 cells, Western blot was performed to detect the protein expression levels of Collagen I, E-cadherin, ACSL4, SPP1, Elastin, Occludin, Vimentin, and α-SMA in the cells, with GAPDH serving as the internal reference (Figures 14B,C). The results showed that compared with the control group, the expression of pro-fibrotic proteins was significantly upregulated in the model group (BLM): the protein levels of Collagen I, Elastin, MMP1, Vimentin, and α-SMA were all markedly increased (P < 0.01). Meanwhile, the expression of SPP1 and ACSL4 was also significantly elevated (P < 0.01), indicating the activation of oxidative stress and the ferroptosis pathway. In contrast, the expression of epithelial barrier-related proteins E-cadherin and Occludin was significantly downregulated (P < 0.01), suggesting impaired epithelial integrity and the occurrence of epithelial-mesenchymal transition (EMT). After JFG intervention, the expression levels of Collagen I, Elastin, MMP1, Vimentin, and α-SMA were all regulated to varying degrees (P < 0.01). The expressions of SPP1 and ACSL4 were also significantly reduced (P < 0.01), while the expressions of E-cadherin and Occludin were partially restored (P < 0.01). The results indicate that JFG may exert anti-pulmonary fibrosis effects by inhibiting oxidative stress and the EMT process, thereby reducing extracellular matrix deposition. These findings are consistent with expectations and further predict that JFG improves pulmonary fibrosis by acting on SPP1 and MMP1 in MRC5.

## 4 Discussion

IPF is a chronic, progressive, and fatal interstitial lung disease. Its core pathogenesis involves recurrent alveolar epithelial injury in genetically susceptible individuals, triggering abnormal repair responses (Honda et al., 2023; Fujimoto et al., 2015). This regulatory imbalance leads to the aberrant activation and proliferation of fibroblasts and myofibroblasts, resulting in excessive ECM deposition and permanent destruction of lung tissue structure (Glass et al., 2022; Vancheri et al., 2024). The prognosis of IPF is extremely poor, with a median survival of only 2.8–5 years post-diagnosis, a 5-year survival rate of less than 30%, and a mortality rate higher than that of most cancers (Honda et al., 2023). Consequently, it is referred to as “a cancer that is not cancer”. From an epidemiological perspective, there are significant regional variations in the prevalence of IPF worldwide. Multiple systematic reviews and large-scale database analyses have shown that its adjusted prevalence estimates range from 0.33 to 4.51 per 10, 000 persons (i.e., approximately 3.3 to 45.1 per 100, 000) (Hutchinson et al., 2015). The estimated number of IPF patients in China is no less than 500, 000, and with the acceleration of population aging, the disease burden is becoming increasingly severe (Huang et al., 2013).

Currently, the clinical treatment of IPF primarily relies on antifibrotic drugs, including nintedanib, pirfenidone, glucocorticoids, immunosuppressants, and end-stage lung transplantation. Although nintedanib and pirfenidone can reduce the annual decline rate of lung function by approximately 50%, they cannot reverse the fibrotic process and are often accompanied by adverse reactions such as diarrhea, photosensitivity, and hepatotoxicity, with significant individual tolerance differences among patients (Glass et al., 2022; Arshad et al., 2024). Glucocorticoids and immunosuppressants are effective in less than 30% of patients, and long-term use can easily lead to severe side effects such as infections and bone marrow suppression, while being ineffective against established fibrosis. Therefore, their widespread use is no longer recommended (Arshad et al., 2024). Although lung transplantation is the only treatment that can prolong survival in advanced patients, it is limited by donor shortages, high surgical risks, and a median post-transplant survival period of about 4.5 years. Other therapeutic strategies, such as the antioxidant N-acetylcysteine, anticoagulation therapy, and vasoactive drugs, have not demonstrated clear efficacy in clinical trials and may even pose additional risks (Duck et al., 2015).

In recent years, TCM has garnered increasing attention from the academic community in the field of anti-organ fibrosis, with its unique value primarily manifested in the theoretical advantages of holistic regulation, multi-target intervention, and syndrome differentiation and treatment (Vancheri et al., 2024; Hutchinson et al., 2015). Unlike chemical drugs that typically target a single site, TCM compounds, through the synergistic action of multiple active ingredients, can simultaneously regulate several key aspects of the fibrosis process (Huang et al., 2013). With the integration of modern molecular biology, network pharmacology, and multi-omics analysis, the material basis and mechanisms of TCM in anti-fibrosis are being gradually revealed. In this context, the present study integrates bioinformatics prediction with experimental validation to investigate the therapeutic potential and underlying mechanisms of JFG, a traditional Chinese medicine formula, in the treatment of IPF.

Through bioinformatics analysis, we systematically investigated the potential mechanisms of JFG in treating IPF. By integrating differentially expressed genes, WGCNA module genes, and predicted JFG targets, we identified 57 overlapping genes that may link JFG to IPF. Network analysis revealed that several of these genes, including FN1, MMP9, MMP1, SPP1, TIMP1, and TOP2A, have been previously associated with the fibrotic process. For instance, FN1 is involved in fibroblast activation and tissue remodeling (Chen et al., 2025), MMP9 and MMP1 play roles in basement membrane destruction and abnormal repair (Ren et al., 2022; Zhang et al., 2025), and SPP1 promotes myofibroblast differentiation and ECM deposition. To validate the relevance of these computationally identified targets, we performed *in vitro* experiments in TGF-β1-stimulated MRC-5 fibroblasts. Western blot analysis confirmed that JFG treatment significantly downregulated the protein expression of SPP1 and MMP1 in a dose-dependent manner (Figures 4C,D), providing experimental evidence supporting the bioinformatics predictions. Additionally, this study identified targets such as BIRC5, CCNA2, and TYMS, which have received less attention in IPF, suggesting that JFG may also regulate non-classical pathways such as cell proliferation and apoptosis (Xia et al., 2025).

GO and KEGG pathway enrichment analyses revealed the potential targets of JFG action at the levels of molecular function and signaling pathways. The analyses indicated that the core target genes were significantly enriched in key biological processes such as cell cycle regulation (e.g., chromosome segregation, G2/M phase transition) (Guo et al., 2024) and dynamic remodeling of the extracellular matrix (ECM) (e.g., collagen metabolism) (Briggs, 2005). This suggests that JFG may influence the fibrotic process by modulating abnormal proliferation and differentiation of epithelial cells and fibroblasts. Consistent with this prediction, our *in vitro* experiments demonstrated that JFG treatment dose-dependently inhibited TGF-β1-induced fibroblast proliferation (CCK-8 assay), migration (wound healing assay), and collagen secretion (hydroxyproline assay). Furthermore, KEGG analysis showed that these genes are involved in pathways such as IL-17, p53, NF-κB, HIF-1, and PI3K-Akt, which collectively regulate inflammation, cellular senescence, hypoxia responses, and ECM deposition in IPF (Wang et al., 2024). *In vivo*, JFG treatment significantly reduced the levels of pro-inflammatory cytokines IL-6 and IL-1β while increasing the anti-inflammatory cytokine IL-10 in the lung tissues of bleomycin-induced fibrotic mice.

This study employed five machine learning algorithms (LASSO, RF, SVM-RFE, NEAT, and KNN) to systematically screen the candidate gene sets derived from multi-source intersections. Through a multi-model integration strategy, we identified four overlapping genes: SPP1, MMP1, AKR1B10, and HTR2A, which may play significant roles in IPF. To validate the relevance of these computationally prioritized targets, we performed Western blot analysis in TGF-β1-stimulated MRC-5 cells and found that JFG treatment dose-dependently downregulated the protein expression of SPP1 and MMP1. These results provide experimental support for the machine learning-based target prioritization. Both SPP1 and MMP1 are closely related to ECM homeostasis. As an extracellular matrix-associated protein, SPP1 is upregulated during pulmonary fibrosis and involved in regulating cell migration and ECM remodeling (Zhang et al., 2025). MMP1 plays a dual role in tissue repair and fibrosis by degrading ECM components, and its abnormal expression is associated with IPF progression. Our finding that JFG suppresses SPP1 and MMP1 expression aligns with its inhibitory effects on collagen deposition observed both *in vitro* (hydroxyproline assay) and *in vivo* (HE staining and hydroxyproline content in mouse lung tissues).

AKR1B10, a member of the aldo-keto reductase family, regulates lipid metabolism and oxidative stress (Li et al., 2024; Brijendra and Niyati, 2022). Studies have shown that metabolic reprogramming, including enhanced glycolysis and inhibited fatty acid oxidation, is a feature of pulmonary fibrosis (Machaliński et al., 2021). JFG may influence lipid metabolism and redox status by regulating AKR1B10. Preliminary evidence supporting this notion comes from our observation that JFG treatment downregulated the expression of ACSL4, a key regulator of ferroptosis and oxidative stress, suggesting a potential role in counteracting oxidative damage. HTR2A, a serotonin receptor, may influence fibroblast function through G protein-coupled signaling. The enrichment of the IL-17 signaling pathway further suggests that JFG may reduce neutrophil infiltration and pro-fibrotic factor release by inhibiting IL-17-mediated inflammatory responses (Wang et al., 2024). Notably, the enrichment of cell cycle-related processes suggests that JFG might affect abnormal cell proliferation and apoptosis escape (Guo et al., 2024; Briggs, 2005). Future studies employing specific inhibitors or genetic knockdown models are needed to establish causal relationships between these predicted targets and the therapeutic effects of JFG.

Single-gene level GSEA further delineated potential pathological axes associated with SPP1, MMP1, AKR1B10, and HTR2A in IPF. SPP1 and AKR1B10 were enriched in pathways regulating epithelial repair and ciliary function, such as axoneme assembly, WNT signaling, and vascular permeability regulation—suggesting their potential roles in maintaining epithelial integrity. Consistent with this, our Western blot analysis showed that JFG treatment upregulated the epithelial marker E-cadherin and downregulated the mesenchymal marker Vimentin in TGF-β1-stimulated cells, indicating a potential role in reversing epithelial-mesenchymal transition (EMT). HTR2A and MMP1 were associated with immune-inflammatory pathways, as evidenced by their enrichment in antimicrobial peptide production, MHC class II molecule-mediated antigen presentation, and complement activation. This aligns with our *in vivo* finding that JFG treatment modulated the levels of inflammatory cytokines (IL-6, IL-1β, IL-10) in fibrotic mouse lungs. GSEA also revealed an oxidative stress signature associated with AKR1B10 (aldehyde detoxification) and SPP1 (hemoglobin complex formation) (Wan et al., 2025), positioning oxidative damage as a potential mechanistic bridge connecting epithelial dysfunction and inflammatory activation with fibrosis progression. Preliminary experimental support for this hypothesis comes from our observation that JFG downregulated ACSL4, a key enzyme in ferroptosis and oxidative stress pathways. The enrichment of these genes in epithelial, immune, and oxidative stress pathways suggests a multi-component network potentially involved in IPF pathogenesis (Qin et al., 2023).

Consistent with established IPF pathology, immune infiltration analysis revealed that fibrotic lung tissues were characterized by an increased enrichment of M2 macrophages and activated fibroblasts, alongside a reduction in plasma cells, CD8^+^ T cells, and NK cells (Epstein et al., 2017; Wu et al., 2025). M2 macrophages, a primary source of pro-fibrotic TGF-β, and activated fibroblasts, the key executors of excessive ECM deposition, are central to this process (Epstein et al., 2017; Gao et al., 2025). Simultaneously, the reduction in plasma cells and T cell subsets also suggests the presence of an immunosuppressive or immune response dysregulated microenvironment in IPF (Isshiki et al., 2023).

Spearman correlation analysis linked the four candidate genes to specific immune cell types. Endothelial cells, fibroblasts, and hematopoietic stem cells were negatively correlated with all four genes, whereas B cells, plasma cells, and class-switched memory B cells showed positive correlations. This suggests a potential role for SPP1 and MMP1 in immune-mediated fibrosis. In line with this, SPP1 is well-documented to promote M2 macrophage polarization and collagen deposition (Wu et al., 2021b; Fei et al., 2025). While our study did not directly quantify immune cell populations, the observed reduction in pro-inflammatory cytokines (IL-6, IL-1β) and increase in anti-inflammatory IL-10 following JFG treatment *in vivo* provide indirect evidence supporting these predicted immunomodulatory effects. Future studies employing flow cytometry or immunohistochemical staining are required to directly validate these findings.

To assess the stability of predicted protein-ligand interactions, molecular dynamics simulations were performed on four complexes: HTR2A-Ammidin, AKR1B10-isorhamnetin, SPP1-quercetin, and MMP1-isorhamnetin. RMSD analysis indicated that all systems reached a stable state (<2 Å) during the simulation. RMSF analysis showed relatively minor conformational fluctuations, suggesting structural stability of the complexes. Solvent accessible surface area (SASA) decreased and stabilized after 60 ns, and the number of hydrogen bonds remained stable throughout the simulation (Figure 11D). The radius of gyration (Rg) remained stable between 2.3 and 2.5 nm, indicating compact folding of the complexes (Yuan and Fu, 2021), but also aligns with the molecular basis of its reported antifibrotic effects, such as inhibiting the TGF-βpathway, and anti-inflammatory effects, such as inhibiting the NF-κB pathway (Dai et al., 2024; He et al., 2023).

Binding free energies were calculated using the MM/PBSA method to quantitatively assess binding affinity. The results showed that all four complexes exhibited favorable binding free energies: AKR1B10-isorhamnetin (ΔG = −127.44 kJ/mol), MMP1-isorhamnetin (ΔG = −120.55 kJ/mol), HTR2A-Ammidin (ΔG = −117.93 kJ/mol), and SPP1-quercetin (ΔG = −73.25 kJ/mol) (Supplementary Figure S3). Energy decomposition analysis revealed that van der Waals interactions contributed substantially to the binding, particularly for MMP1-isorhamnetin.By integrating molecular docking with molecular dynamics simulations and MM/PBSA binding free energy calculations, we have advanced from static binding prediction to quantitative dynamic assessment. These computational analyses suggest that active components in JFG, such as flavonoids and phytosterols, may form stable complexes with core targets including HTR2A, AKR1B10, SPP1, and MMP1, providing a theoretical basis for their potential interactions.

In summary, this study integrates computational predictions with experimental validation to investigate the therapeutic potential of JFG in IPF. Bioinformatics analysis identified SPP1, MMP1, AKR1B10, and HTR2A as candidate biomarkers and potential targets of JFG. Molecular dynamics simulations predicted stable interactions between JFG components and these targets, with favorable binding free energies. Experimental validation demonstrated that JFG attenuates bleomycin-induced pulmonary fibrosis in mice, as evidenced by reduced pathological damage, decreased collagen deposition (Masson’s trichrome staining and hydroxyproline content), downregulation of α-SMA and Collagen I (IHC), and modulation of inflammatory cytokines (IL-6, IL-1β, IL-10). *In vitro*, JFG inhibited TGF-β1-induced fibroblast proliferation, migration, and collagen secretion, and dose-dependently downregulated the expression of SPP1, MMP1, α-SMA, Collagen I, Vimentin, and ACSL4, while upregulating E-cadherin and Occludin. These results suggest that JFG may exert anti-fibrotic effects through modulation of fibrotic pathways, EMT, oxidative stress, and inflammatory responses.

However, several limitations of this study should be acknowledged. First, although we have validated the expression changes of SPP1 and MMP1 following JFG treatment, direct biochemical evidence of JFG components binding to these targets (e.g., surface plasmon resonance, cellular thermal shift assay) is lacking. Second, the immunomodulatory effects predicted by immune infiltration analysis require validation using flow cytometry or immunohistochemical staining of immune cell markers. Third, pharmacokinetic studies are needed to determine whether JFG components reach therapeutic concentrations in lung tissue. Fourth, genetic approaches (e.g., siRNA knockdown of individual targets) are needed to establish causal relationships between specific targets and the therapeutic effects of JFG.

Future directions include: (1) validating direct interactions between JFG components and core targets using biophysical methods; (2) employing genetic gain- and loss-of-function studies to establish causal relationships; (3) investigating immunomodulatory effects through detailed immunophenotyping; (4) conducting pharmacokinetic studies to assess lung tissue exposure; and (5) exploring the therapeutic potential of JFG in additional animal models and ultimately in clinical settings.

## 5 Research highlights

This study, for the first time, systematically reveals the multi-target therapeutic mechanism of the traditional Chinese medicine compound JFG in synergistically regulating the immune microenvironment of pulmonary fibrosis and abnormal cell proliferation by acting on core targets such as SPP1 and MMP1, through the integration of bioinformatics and multi-omics analysis combined with machine learning.

## Data Availability

The datasets presented in this study can be found in online repositories. The names of the repository/repositories and accession number(s) can be found in the article/Supplementary Material.
