# Supplementary material for: SPP1 and MMP1 as key therapeutic targets of Jingfang Granule in idiopathic pulmonary fibrosis: integrated bioinformatics and machine learning analysis
Source: Front Pharmacol. 2026 Jun 10;17:1739181. doi: 10.3389/fphar.2026.1739181 (PMC13291481; doi:10.3389/fphar.2026.1739181)
Supplement: Supplementary file 3 [file DataSheet3.docx]

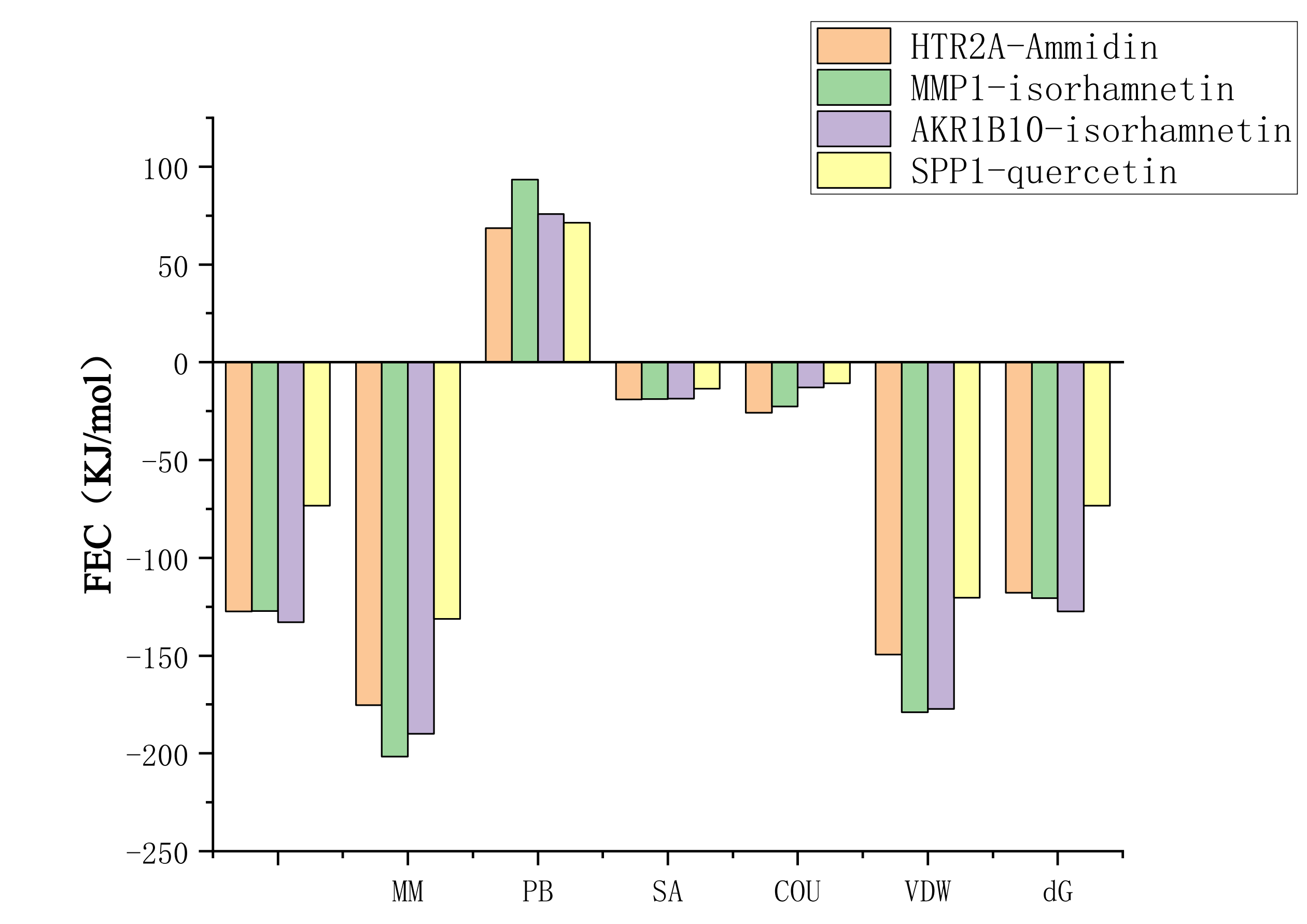


**Supplementary FIGURE** 3 Binding free energy decomposition diagram of four receptor-ligand complexes calculated based on MM/PBSA method.

notes: The horizontal axis represents different energy terms (MM: molecular mechanical energy; PB: polar solvation energy; SA: non-polar solvation energy; COU: Coulomb/electrostatic interaction; VDW: van der Waals interaction; dG: total binding free energy).
