# Supplementary figures and images for: SPP1 and MMP1 as key therapeutic targets of Jingfang Granule in idiopathic pulmonary fibrosis: integrated bioinformatics and machine learning analysis
Source: Front Pharmacol. 2026 Jun 10;17:1739181. doi: 10.3389/fphar.2026.1739181 (PMC13291481; doi:10.3389/fphar.2026.1739181)

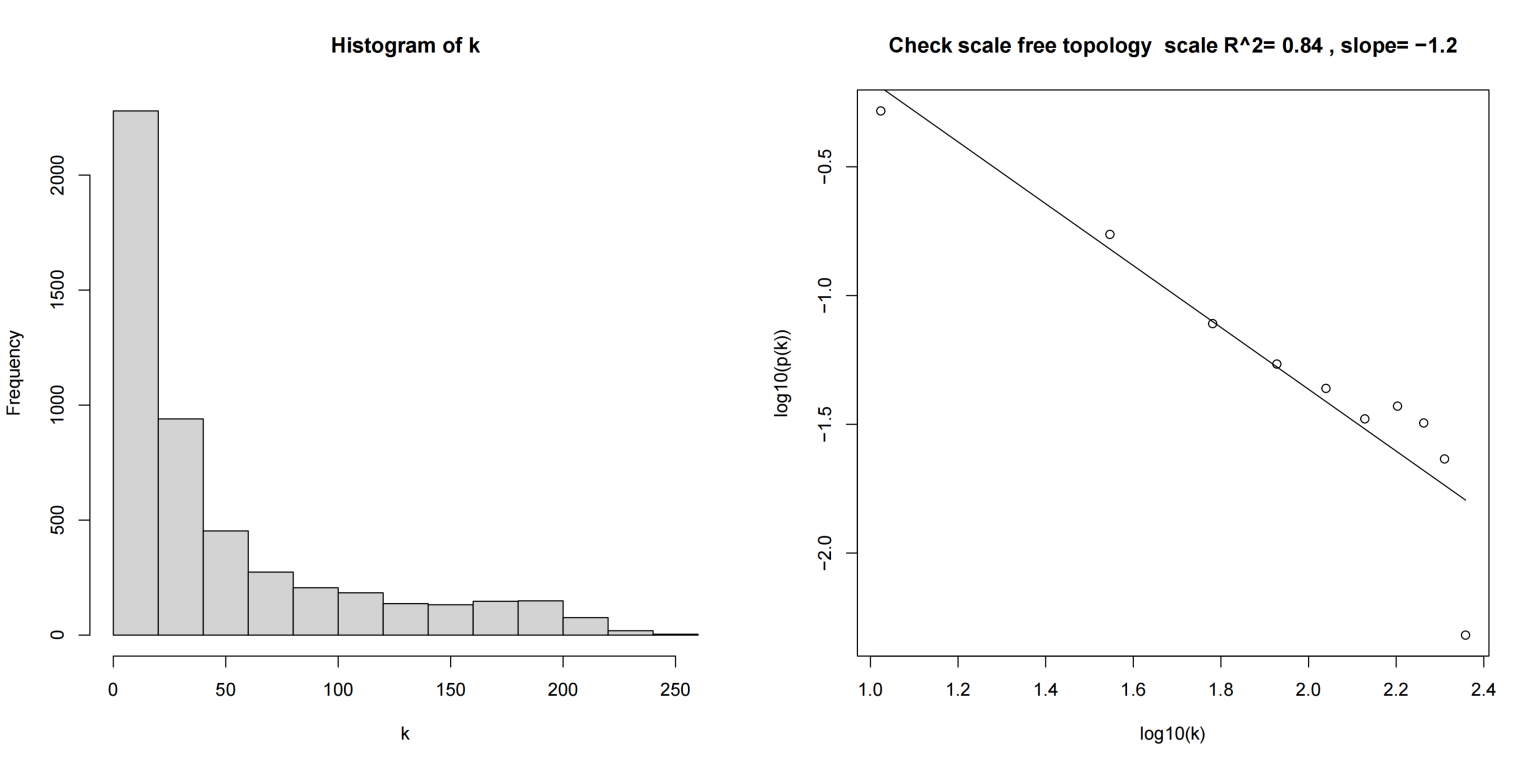


**Supplementary FIGURE** 2 Degree distribution plot in double logarithmic coordinates.

Supplement: Supplementary file 5 [file DataSheet2.docx]

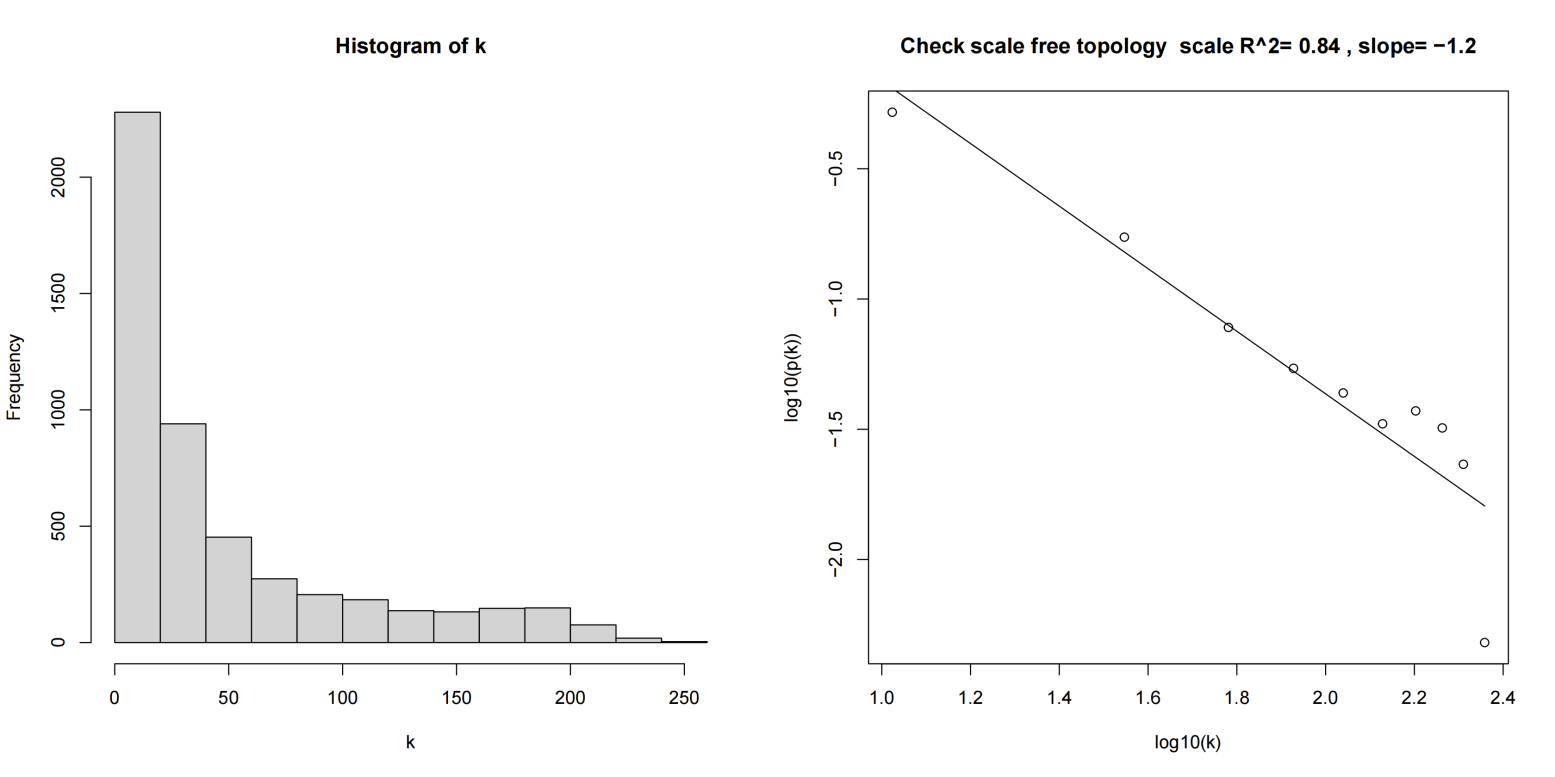


**Supplementary FIGURE** 1 Degree distribution histogram.

Supplement: Supplementary file 6 [file DataSheet1.docx]
